# Prevalence of unintended pregnancy and associated factors among married women in west Belessa Woreda, Northwest Ethiopia, 2016

**DOI:** 10.1186/s12978-018-0649-6

**Published:** 2018-12-07

**Authors:** Adino Tesfahun Tsegaye, Menderie Mengistu, Alemayehu Shimeka

**Affiliations:** 1 Department of Epidemiology and Biostatistics, Institute of Public Health, College of Medicine and Health Sciences, University of Gondar, Gondar, Ethiopia; 2 University of Gondar Referral Hospital, University of Gondar, Gondar, Ethiopia

**Keywords:** Unintended pregnancy, Married women, Mistimed pregnancy, Unwanted pregnancy, Ethiopia

## Abstract

**Background:**

Unintended pregnancies can have adverse physical, mental, social, and economic outcomes. Illegal abortions and associated complications often follow unintended pregnancies and claim the lives of many women in developing countries. To better understand how unintended pregnancy impacts married women, this study aimed to assess the prevalence of unintended pregnancies and associated factors among married pregnant women in West Belessa woreda, Ethiopia.

**Methods:**

A community-based cross-sectional study was conducted from August to September 2015.. A multistage stratified sampling technique was used to select nine kebeles, to participate in the study. A total of 619 married pregnant women were selected from these kebeles by the simple random sampling technique and data were collected with a structured questionnaire. Binary logistic regression analysis was used to identify factors associated with the unintended pregnancies. A *p*-value of < 0.05 in the multi-variable model was used to identify significance.

**Result:**

A total of 592 married pregnant women were surveyed regarding their intention to become pregnant. The prevalence of unintended pregnancy was 13.7%. Age at pregnancy (AOR: 15.2, 95% CI (1.9, 125.2)), history of stillbirth (AOR: 3.3, 95% CI (1.4, 7.9)), discussing pregnancy related issues with husbands (AOR: 2.3, 95% CI (1.1, 5.0)), making family planning decisions on their own (AOR: 0.4, (0.2, 0.8)), and making family planning decisions with their husbands (AOR: 95% CI 0.2 (0.1, 0.4)) were significantly associated with unintended pregnancies in this group.

**Conclusion:**

The magnitude of unintended pregnancy in the study area was low. Age at pregnancy, history of stillbirth and involvement of partners in making reproductive health choices were associated with unintended pregnancies. Empowering women to make family planning decisions and increasing partner involvement in reproductive health could decrease unintended pregnancies**.**

## Plain English summary

Unintended pregnancies are a major public health problem that can have adverse physical, mental, social, and economic outcomes. In this study, we observed the burden of unintended pregnancy among married women via a community-based survey of pregnant women. Eight rural kebeles and one urban kebele were included. The list of married pregnant women from each kebele was used as a sampling frame.

Participants were selected randomly and asked about their socio-demographic, reproductive and environmental characteristics. Additionally, the classification of their current pregnancy as intended or unintended was assessed and further questions were recorded among women who reported their current pregnancy to be unintended.

The total sample size was 619 participants. However, only 592 participants responded to the questionnaire. Out of the respondents, 523 were living in rural kebeles and 378 of the participants were unable to read and write. The mean age of the respondents was 28.4 years (±7.03). Out of the 592 women, 81 had unintended pregnancies and out of 81 women who had unintended pregnancies, 60 were mistimed and 21 were unwanted.

Regarding the determinants of unintended pregnancies, older age, history of stillbirth, and not discussing pregnancy with husbands were factors which can increase the likelihood of unintended pregnancies. Meanwhile, making a family planning decision alone and discussing family planning with their husbands had a negative correlation and thus a protective effect in developing unintended pregnancies.

## Background

Unintended pregnancy is a major global concern due to its association with adverse physical, mental, social and economic outcomes. It is not just a problem of young, poor women or minorities; it affects all segments of the community and contributes greatly to maternal and infant mortality [1]. Out of the 210 million annual pregnancies worldwide, about 79 million are unintended and out of this, 50% end in abortion [2].

Globally, the maternal mortality ratio (MMR) was 216 maternal deaths per 100,000 live births in 2015. Developing countries account for approximately 99% of the global maternal deaths in 2015, with sub-Saharan Africa alone accounting for roughly 66% [3]. The 2016 Ethiopian Demographic and Health Survey (EDHS) report showed the MMR in Ethiopia was 412 per 100,000 live births [4].

Between 20 and 40% of all births that occur in developing countries are unwanted, posing a hardship for families and jeopardizing the health of millions of women and children [3]. Women who experience an unintended pregnancy are less likely to have prenatal, perinatal and postnatal care [5].

Unintended pregnancies can result from a lack of contraceptive use, contraceptive failure and incorrect use of contraceptives [5]. Multiple studies have also shown other variables that influence the likelihood of unintended pregnancies including sociodemographic and economic conditions [6–12] as well as reproductive and environmental factors [6, 7, 9, 11–13].

An institution based study conducted in Felege Hiwot hospital in Ethiopia’s Amhara Region showed a prevalence of unintended pregnancies at 29.5% [9] and another study conducted in Arba Minch, southern Ethiopia, showed a prevalence of unintended pregnancies of 19.4% [14]. Most of the studies conducted in Ethiopia regarding unintended pregnancies are institution based and community-based information on unintended pregnancies among married women is limited. Therefore, this study aimed to assess the status of unintended pregnancies among married pregnant women in the general population. This study may inform policymakers in addressing the burden of unintended pregnancies and related complications among married women.

## Methods

### Study design and setting

A community-based cross-sectional study was conducted to assess unintended pregnancy and associated factors among married pregnant women in West Belessa Woreda, Northwest Ethiopia, from August to September 2015. West Belessa Woreda is in North Gondar Zone, Amhara National Regional State. The Woreda is located 784 km northwest of Addis Ababa, the capital of Ethiopia and 207 km from Bahir Dar, the regional capital. The Woreda has eight public health centers, 30 health posts, and two private clinics. Eighty-five percent of the population has access to health services. The district has ten health officers, three environmental health officers, 53 nurses, 12 pharmacy technicians, two pharmacists, six laboratory technicians and a technologist, two urban health extension workers, and 65 rural health extension workers that support the health system in the Woreda [15].

### Participants

The study population consisted of married pregnant women living in West Belessa Woreda. Participants were selected randomly and married pregnant women who lived in the study area for at least 6 months were included and women who were seriously ill during the data collection period were excluded.

The sample size was determined using a single population proportion formula considering the following assumptions of the prevalence of unintended pregnancy 26% [16], 95% confidence level, Z^2^_α*/2*_ = 1.96, and margin of error 5%.

$$ {\displaystyle \begin{array}{l}\mathrm{n}=\frac{{\left(\mathrm{Z}\infty /2\right)}^2\times \mathrm{P}\left(1\hbox{-} 2\right)}{{\mathrm{d}}^2}=\frac{(1.96)^2\times 0.26\left(1-0.26\right)}{0.05^2}\\ {}\mathrm{n}=295\end{array}} $$

**Fig. 1:**
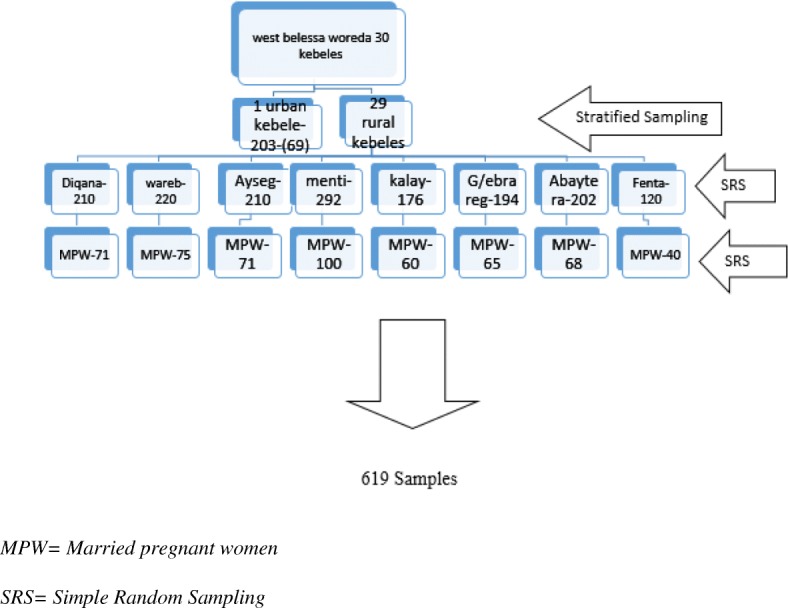
Schematic presentation of sampling procedures

With a design effect of 2 and a 5% non-response rate, the total sample size needed was 619. A multistage stratified sampling technique was used to select the participants. According to information received from the Woreda health office, on average there were 100 to 300 pregnant women in each kebele (the smallest administrative unit). From 30 kebeles stratified into two (29 rural kebeles and one urban kebele), eight rural kebeles were selected by simple random sampling technique using the lottery method and the urban kebele was directly taken. The final sample size was distributed to each selected kebele proportional to the number of married pregnant women present (Fig. 1). A list of married pregnant women was provided by Health Extension Workers (HEW) working in the area and pregnant women were selected through a simple random sampling technique using computer-generated random numbers.

### Variables of the study

The dependent variable was unintended pregnancy. The independent variables were the following: socio-demographic factors (age, educational status, family size, age at first marriage); institutional and organizational factors (supervision by HEW, supervision by Health Development Army (HDA), provision of family planning (FP), availability of transportation, delivery services); environmental and behavioral factors (distance of health facilities, accessibility of health facilities, knowledge of contraceptives, decision to use family planning, communication with husband about pregnancy), and maternal factors (comorbidity, gravidity, desired number of children, history of previous birth, power to decide on pregnancy, FP method utilization). Unintended pregnancy was defined as a pregnancy that was either unwanted or mistimed.

### Data collection tools and procedures

Data were collected by interviewing married pregnant women using a standardized structured questionnaire. The questionnaire was first prepared in English and then translated into the local language Amharic and then translated back to English by a third party to check its consistency and conceptual equivalence. The Amharic version was pre-tested outside the study area. Trained clinical nurses collected the data under the supervision of trained public health professionals. The collected data were checked for completeness and consistency on a daily basis.

### Data processing and analysis

Data entry, cleaning, and coding were done using Epi-Info version 7 and exported to SPSS version 20 for further analysis. Descriptive and summary statistics were done. Binary logistic regression analysis was used to identify determinants of unintended pregnancies. Variables which had a *p*-value of <=0.2 in the bivariable analysis were subsequently entered into a multivariable logistic regression analysis and variables with a *p*-value of < 0.05 in the multivariable model were identified as statistically significant.

## Result

### Socio-demographic characteristics

Overall, 592 married women were interviewed with a response rate of 95.6%. Out of the total respondents, 92.1% (545) were Orthodox Christians and 7.9% (47) were Muslims. Eighty-eight percent (523) of respondents were living in rural kebeles and 63.9% (378) were unable to read and write. The mean age of respondents was 28.4 years (±7.03). Distance to nearby health facilities varied with 42.4% (251) living a walking distance of 30 min or less from a health facility, and 22.8% (135) living two or more hours away by foot (Table 1).

**Table 1:** Socio-demographic characteristics of West Belessa Woreda*,* 2016 (*N* = 592)

Variable	Frequency	Percentage
Religion		
Orthodox	545	92.1
Muslim	47	7.9
Ethnicity		
Amhara	583	98.5
Tigrie	6	1
Agew	3	0.5
Residence		
Rural	523	88.3
Urban	69	11.7
Age		
15–19	63	10.6
20–24	122	20.6
25–29	154	26
30–34	128	21.6
35–39	81	13.7
40–44	32	5.4
45–49	12	2
Educational status of women		
Unable to read and write	378	63.9
Read and write	153	25.8
Primary education	47	7.9
Secondary education	14	2.4
Partner’s educational status		
Unable to read and write	366	61.82
Read and write	152	25.68
Primary education	47	7.94
Secondary education and above	27	4.56
Distance from health facility		
< 30 min	251	42.4
1 h	206	34.8
≥ 2 h	135	22

### Reproductive factors

Most women surveyed were multi-gravida and 42.4% (251) had five or more prior pregnancies. More than 98% (588) of participants had awareness about contraceptive methods and 53.5% (317) had ever used contraceptives. Regarding home to home supervision by community health advocates, 70.8% (419) were supervised by health extension workers and 48.1% (285) were supervised by members of the health development army (Table 2).

**Table 2:** Reproductive characteristics of pregnant women in West Belessa Woreda, 2016 (*N* = 592)

Variable	Frequency	Percentage
Gravidity		
≤ 2	164	27.7
3–4	177	29.9
≥ 5	251	42.4
Married life		
< 5 years	120	20.27
5–10 Years	183	30.91
> 10 Years	289	48.82
Parity		
≤ 1	86	14.5
1–2	162	27.4
3–4	187	30.6
≥ 5	163	27.5
No. of live children		
None	87	14.7
1–2	168	28.4
3–4	180	30.4
≥ 5	157	26.5
Baby with disability		
Yes	19	3.2
No	573	96.8
History of still birth		
Yes	39	6.6
No	553	93.4
Ever used contraceptives		
Yes	275	46.5
No	317	53.5
Family planning decision maker		
Husband	122	20.6
Myself	142	24
Both	328	55.4
Discussion with husband about FP		
yes	540	91.2
No	52	8.8

### Prevalence of unintended pregnancies

**Fig. 2:**
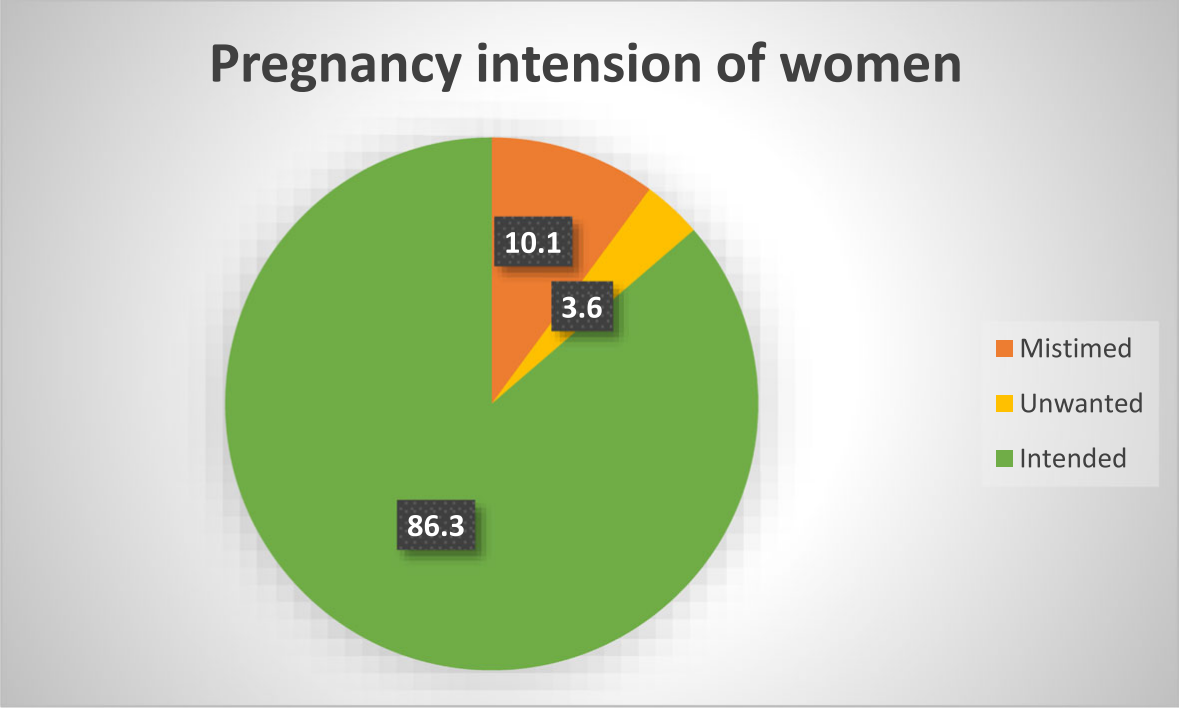
Pregnancy intention among married pregnant women in West Belessa Woreda, 2016

Out of 592 respondents, 13.7% (81) women reported that their pregnancies were unintended, while the rest 86.3% had intended pregnancies. Out of 81 women who had unintended pregnancies, 74.1% (60) were mistimed and 25.9% [17] were unwanted (Fig. 2).

The reasons for unintended pregnancies were, poor economic capacity (25.9% [17]), husband’s influence (25.9% [17]), having enough children (19.8% [16]), and rape (28.4% [18]).

### Determinants of unintended pregnancies

In the multivariable logistic regression analysis, history of stillbirth, age, decision making on family planning, and discussing pregnancy related issues with husbands had a statistically significant association with unintended pregnancy (Table 3).

**Table 3:** Multivariable analysis of predictors of unintended pregnancy in west Belessa woreda, 2016

Variables	Pregnancy intention frequency (percentage)				
		Unintended	Intended	COR (95% CI)	AOR (95% CI)
History of still birth					
Yes		15 (38.5)	24 (61.5)	4.6 (2.3, 9.2)	3.3 (1.4, 7.9)
No		66 (11.9)	487 (88.1)		
Gravidity					
≤2		16 (9.8)	148 (90.2)	1	1
3–4		17 (9.6)	160 (90.4)	0.9 (0.5, 2.0)	2.3 (0.5, 10.3)
≥5		48 (19.1)	203 (80.9)	2.2 (1.2, 4.0)	1.8 (0.2, 12.4)
Parity					
None		12 (13.95)	74 (86.05)	1	1
1–2		13 (8.0)	149 (92.0)	0.5 (0.2, 1.2)	0.4 (0.1, 1.5)
3–4		15 (8.3)	166 (91.7)	0.6 (0.2, 1.2)	0.3 (0.04, 1.5)
≥ 5		41 (25.1)	122 (74.9)	2.1 (1.0, 4.2)	0.4 (0.1, 3.7)
Age					
15–19		9 (14.3)	54 (85.7)	1	1
20–24		10 (8.2)	112 (91.1)	0.5 (0.2, 1.4)	1.4 (0.4, 4.7)
25–29		14 (9.1)	140 (90.9)	0.6 (0.2, 1.5)	1.8 (0.4, 8.5)
30–34		14 (10.9)	114 (89.1)	0.7 (0.3, 1.8)	1.8 (0.3, 12.4)
35–39		13 (16.1)	68 (83.9)	1.1 (0.4, 2.9)	2.7 (0.4, 20.1)
40–44		15 (46.9)	17 (56.1)	5.3 (2.0, 14.2)	15.2 (1.9, 125.2)
45 = 49		6 (50.0)	6 (50.0)	6.0 (1.6. 22.8)	8.2 (0.8, 81.4)
Length of married life					
< 5 years		14 (11.7)	106 (88.3)	1	1
5–10 Years		17 (9.3)	166 (90.7)	0.8 (0.4, 1.6)	0.8 (0.2, 3.4)
> 10 Years		50 (17.3)	239 (82.7)	1.6 (0.8, 3.0)	0.5 (0.1, 3.2)
Baby with disability					
Yes		10 (27.8)	26 (72.2)	2.6 (1.2, 5.7)	2.3 (0.8, 6.1)
No		71 (12.8)	485 (87.2)	1	1
Walking distance from health facility					
< 30 min		28 (11.2)	223 (88.8)	1	1
30 min-1 h		22 (10.7)	184 (89.3)	0.9 (0.5, 1.7)	0.7 (0.4, 1.4)
≥2 h		31 (23.0)	104 (77.0)	2.4 (1.4, 4.2)	0.7 (0.4, 1.5)
Discussing pregnancy related issue with husband					
Yes		63 (11.7)	477 (88.3)	1	1
No		18 (34.6)	34 (65.4)	4.0 (2.1, 7.5)	2.3 (1.1, 5.0)
Ever used contraceptive					
Yes		27 (9.8)	248 (90.2)	1	1
No		54 (17.0)	263 (83.0)	1.9 (1.2, 3.1)	1.1 (0.6, 2.0)
Decision on family planning					
Husband		38 (0.3)	84 (0.7)	1	1
My self		20 (0.1)	122 (0.9)	0.4 (0.2, 0.7)	0.4 (0.2, 0.8)
Both		23 (0.1)	305 (0.9)	0.2 (0.1, 0.3)	0.2 (0.1, 0.4)

The odds of unintended pregnancy among married pregnant women who had a history of stillbirth was 3.3 times (AOR: 3.3, 95% CI (1.4, 7.9)) higher than those without a history of stillbirth. The odds of unintended pregnancies among women in the age group 40–44 was 15 times higher (AOR: 15.2, 95% CI (1.9, 125.2)) than those in the age group of 15–19. Women who do not discuss their pregnancy with their husbands had 2.3 times (AOR: 2.3, 95% CI (1.1, 5.0)) higher odds of having unintended pregnancies than women who did communicate. The odds of unintended pregnancy among women who chose to use family planning by themselves was 60% (AOR: 0.4, (0.2, 0.8)) less than those who followed only their husbands’ decision. The odds of unintended pregnancies among women who decided to use a family planning method in conjunction with their husbands’ was 80% (AOR: 95% CI 0.2 (0.1, 0.4)) less than those who followed only their husbands’ decision.

## Discussion

This study aimed to measure the magnitude of unintended pregnancies and associated factors. According to this study’s finding, the prevalence of unintended pregnancy was 13.7%. This result is lower than studies done in Southern Ethiopia (Arba Minch) [14], the Amhara region (Felege Hiwot Hospital [9], and Western Ethiopia (Ganji) [10]. The majority of this study’s participants consisted of rural women who traditionally bear many children [19]. Moreover, the participants of this study are married women who, as evidenced by other studies done in Ethiopia, are less likely to have unintended pregnancies [20, 21]. In addition, the Ethiopian government is striving to address maternal health problems and there have been improvements in access and utilization of reproductive health care services over time [4, 19, 22]. When we compare this result with the study done in Ganji, the health service coverage in Ganji was 52% [10] while the health service coverage for this study was 85%. Therefore, the growing availability of family planning services in the study communities could explain the lower rate of unintended pregnancies.

In this study, a history of stillbirth was significantly associated with unintended pregnancy. When a woman had a history of stillbirth, she might fear its re-occurrence and have less desire to become pregnant again [17, 23]. Unintended pregnancy was also higher among married pregnant women in the age group of 40–44 years. This finding is consistent with a study conducted in Ganji woreda [10]. Women above the age of forty are less likely to desire further pregnancies since they likely have the ideal number of children that they want. Moreover, complications related to pregnancy are very common in this age group which may further deter them from desiring pregnancy [18, 24]. Additionally, this group of women are older and less literate and may have less knowledge about the importance and accessibility of reproductive health services [25]. Spousal support among this group may also be limited. Relationships in this age group are more likely to be unstable and women may perceive themselves as having a low risk of conceiving contraceptives [26]. Also, they are more likely to have past experiences of unwanted contraceptive side effects that may deter their use of contraceptives [26, 27]. This combination of characteristics may make these women less likely to use family planning methods correctly [28] and more likely to practice unsafe sex. In addition, some diseases associated with previous pregnancies may influence their interest in becoming pregnant.

The role of partners has a significant effect on a woman’s intention to become pregnant. Women who did not have open communication about their pregnancy with their husbands had higher odds of having an unintended pregnancy than women who did communicate. When women have open communication about their pregnancy before and after conception, they get the support of their partners and have a higher likelihood of planning a pregnancy. In the absence of communication with their husbands, rural women, who spend most of their time at home, may not have adequate information about family planning which may result in unintended pregnancies. Moreover, women might not have the autonomy to decide about their fertility [29].

There appears to be a protective effect from unintended pregnancies when both husband and wife discuss and agree on their family planning goals as well as when a wife makes family planning decisions independently. Unintended pregnancy increases when the husband is the sole decision-maker in the family planning. This result is consistent with a study conducted in Senegal [11]. When women are empowered to be decision-makers and/or have the support of their husband in making family planning decision, they have improved knowledge and access to reproductive health care services [30, 31]. Therefore, joint decision making and/or sole female decision making decreases the occurrence of unintended pregnancies and allows families to lead more stable lives.

Although this study was strengthened by being community-based, the addition of qualitative measures would enrich the data. In addition, social desirability bias could lead women to report that their pregnancy was intended even though it was not, leading to an underestimate of the burden of unintended pregnancies. The other limitation of this study is that only married pregnant women were included. Whereas the majority of the unintended pregnancies result from illegitimate sexual intercourse, which is most common among sexually active teenagers and unmarried women. Lastly, the preference of some women not to disclose their unintended pregnancy might underestimate its burden.

## Conclusion

Our results showed a low rate of unintended pregnancies among married women in the study areas at 13.7%. Age, history of adverse pregnancy outcomes and involvement of partners in reproductive health issues were associated with unintended pregnancies. Women who are in the late reproductive ages need close follow up and support in family planning. Furthermore, psychological support for women who have a history of adverse outcomes of pregnancy such as a history of stillbirth would also be an important group to connect to services to decrease the burden. Moreover, empowering women and increasing partner involvement in reproductive health may decrease unwanted pregnancies and its downstream consequences. Therefore, reproductive health programs that focus on building confidence and autonomy of women in planning their pregnancy may have a high impact on reducing unintended pregnancy.

## Abbreviations

ANRS: Amhara National Regional State
AOR: Adjusted Odds ration
CSA: Central Statics Agency
EDHS: Ethiopian Demography and Health Survey
FMOH: Federal Ministry of Health
HEW: Health Extension Worker
MDG: Millennium Development Goal
MMR: Maternal Mortality Ratio
WHO: World Health Organization
